# Complement Factor B Is a Determinant of Both Metabolic and Cardiovascular Features of Metabolic Syndrome

**DOI:** 10.1161/HYPERTENSIONAHA.117.09242

**Published:** 2017-08-09

**Authors:** Philip M. Coan, Marjorie Barrier, Neza Alfazema, Roderick N. Carter, Sophie Marion de Procé, Xaquin C. Dopico, Ana Garcia Diaz, Adrian Thomson, Lucy H. Jackson-Jones, Ben Moyon, Zoe Webster, David Ross, Julie Moss, Mark J. Arends, Nicholas M. Morton, Timothy J. Aitman

**Affiliations:** From the Centre for Genomic and Experimental Medicine, MRC Institute for Genetics and Molecular Medicine, Edinburgh, United Kingdom (P.M.C., M.B., N.A., S.M.P., X.C.D., D.R., J.M., T.J.A.); British Heart Foundation Centre for Cardiovascular Science, Queen’s Medical Research Institute (P.M.C., M.B., N.A., R.N.C., A.T., L.H.J.-J., N.M.M., T.J.A.) and Royal (Dick) School of Veterinary Studies (X.C.D.), University of Edinburgh, United Kingdom; Department of Medicine (A.G.D., T.J.A) and Embryonic Stem Cell and Transgenics Facility, MRC Clinical Sciences Centre (B.M., Z.W.), Imperial College London, United Kingdom; and Division of Pathology, Centre for Comparative Pathology, Cancer Research UK Edinburgh Centre, United Kingdom (M.J.A.).

**Keywords:** adipose tissue, blood pressure, complement system proteins, glucose, hypertension

## Abstract

CFB (complement factor B) is elevated in adipose tissue and serum from patients with type 2 diabetes mellitus and cardiovascular disease, but the causal relationship to disease pathogenesis is unclear. Cfb is also elevated in adipose tissue and serum of the spontaneously hypertensive rat, a well-characterized model of metabolic syndrome. To establish the role of CFB in metabolic syndrome, we knocked out the *Cfb* gene in the spontaneously hypertensive rat. *Cfb*^−/−^ rats showed improved glucose tolerance and insulin sensitivity, redistribution of visceral to subcutaneous fat, increased adipocyte mitochondrial respiration, and marked changes in gene expression. *Cfb*^−/−^ rats also had lower blood pressure, increased ejection fraction and fractional shortening, and reduced left ventricular mass. These changes in metabolism and gene expression, in adipose tissue and left ventricle, suggest new adipose tissue-intrinsic and blood pressure-independent mechanisms for insulin resistance and cardiac hypertrophy in the spontaneously hypertensive rat. In silico analysis of the human *CFB* locus revealed 2 *cis*-regulated expression quantitative trait loci for *CFB* expression significantly associated with visceral fat, circulating triglycerides and hypertension in genome-wide association studies. Together, these data demonstrate a key role for *CFB* in the development of spontaneously hypertensive rat metabolic syndrome phenotypes and of related traits in humans and indicate the potential for CFB as a novel target for treatment of cardiometabolic disease.

## Abstract 2

Supplemental Digital Content is available in the text.

Metabolic syndrome (MetS) represents a complex clustering of cardiometabolic traits, including hypertension, insulin resistance, glucose intolerance, and dyslipidemia, all of which increase the risk of developing type 2 diabetes mellitus and cardiovascular disease.^1^ Despite established environmental risk factors and genome-wide association study (GWAS) hits that link genetic variation to MetS constituents, the molecular and cellular events underlying its development remain incompletely understood.^2,3^

Chronic low-grade inflammation and innate immune system overactivation are now recognized causes of type 2 diabetes mellitus and MetS.^4,5^ In particular, the alternative pathway (AP) has received attention for its potential causal role in cardiometabolic disease.^6^ AP activation requires CFB (complement factor B) to bind C3 to form C3B, which opsonises pathogens and contributes to the formation of the membrane attack complex.^6^ Thus, CFB is fundamental to pathogen clearance and host cell apoptosis. However, increased circulating CFB has been found in patients with type 2 diabetes mellitus,^7^ and expression of adipose tissue CFB correlates significantly with fasting glucose and circulating lipids.^8^ Elevated circulating CFB has also been found to increase the risk of endothelial dysfunction^9^ and coronary heart disease.^10^

Because of the complex genetic basis of human MetS, the spontaneously hypertensive rat (SHR), which exhibits hypertension, insulin resistance, and dyslipidemia, has been extensively studied as a MetS model.^11–13^

Multiple studies have identified SHR genes associated with features of MetS, many of which show conserved pathologies in humans.^14–17^

The rat *Cfb* gene resides within the major histocompatibility region on chromosome 20p12.^18^ In SHR, this region has been demonstrated to be important in blood pressure regulation,^19^ serum cholesterol, adiposity, and glucose tolerance.^20,21^ In this study, we knocked out *Cfb* in SHR to test the hypothesis that *Cfb* is necessary for the full expression of cardiometabolic pathophysiological traits in this model of MetS.

## Methods

Detailed methods are available in the online-only Data Supplement.

### Rats

*Cfb*^−/−^ rats were generated using SHR/NCrl rats (Charles River, Margate, United Kingdom), by microinjecting Zinc-finger nuclease (ZFN) mRNA (Sigma), targeted to exon 6 of *Cfb* (target sequence: CCCCTCGGGCTCCATGaatatcTACATGGTGCTGGATG), into 1-cell stage SHR/NCrl embryos that were implanted into pseudopregnant rats. Heterozygous progeny, from a founder harboring a 19-base pair deletion in *Cfb*, were intercrossed to homozygosity. A search for off-target events, conducted by whole genome sequencing confirmed the 19-base pair deletion. Six additional putative mutations, analyzed by Sanger Sequencing, were determined to be false positives (Table S1). Rats were housed with free access to food and water. All procedures were performed in accordance with UK Home Office regulations.

### Statistics

Unpaired *t* test or 2-way ANOVA (Minitab Express) were used to assess differences between genotype and treatment. All results are mean±SEM. *P*<0.05 was considered significant.

## Results

### Generation of a *Cfb* Knockout Rat

Using data from a quantitative trait transcript analysis of recombinant inbred strains derived from a SHR×Brown Norway (BN-*Lx*/Cub) cross,^22^ we identified *Cfb* transcript levels as uniquely and strongly correlated significantly across the recombinant inbred strains for metabolically relevant traits (glucose uptake in isolated adipocytes, r^2^=−0.65, *P*_(adj)_=0.0003; basal lipogenesis in epididymal fat, r^2^=−0.64, *P*_(adj)_=0.0002; serum high-density lipoprotein cholesterol, r^2^=−0.64, *P*_(adj)_=0.0005) and significantly differentially expressed in adipose tissue between parental strains (SHR versus Brown Norway, 1.47-fold *P*_(adj)_<0.05). Overexpression in SHR adipose tissue was confirmed by quantitative polymerase chain reaction by comparing a further insulin sensitive/normotensive Wistar Kyoto strain (WKY/NCrl; Figure S1A). *Cfb* was also overexpressed in SHR left ventricle (LV), but not liver, compared with WKY (Figure S1A). *Cfb* overexpression in SHR was associated with increased AP activity compared with WKY (Figure S1B). Analysis of the *Cfb* gene and its adjacent region revealed 14 variants unique to SHR, not present in Brown Norway or WKY; 2 variants reside upstream of the transcription start site (Figure S1C). To investigate the potential causative role of *Cfb* in the cardiometabolic traits of SHR, a 19-base pair deletion in exon 6 of the *Cfb* gene in the SHR germline was made using ZFNs (Figure S1D). Abolition of *Cfb* expression was confirmed by quantitative polymerase chain reaction and immunoblot (Figure S1E), and loss of Cfb function was confirmed by ablation of serum AP activity (Figure S1F).

### Glucose Homeostasis

**Figure 1.**
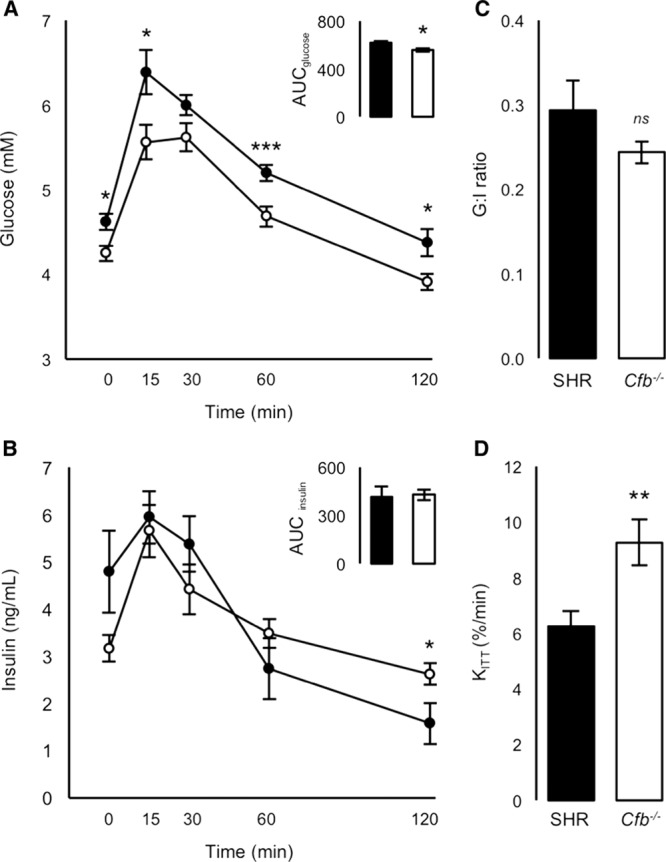
Glucose homeostasis. **A**, Glucose concentration curve during oral glucose tolerance (OGTT; inset, area under the curve, area under the curve (AUC), glucose. **B**, Plasma insulin concentration curve of OGTT (inset; area under the curve insulin). **C**, G:I ratio, (AUC_glucose_:AUC_insulin_). **D**, Insulin-stimulated glucose clearance (K_ITT_). Spontaneously hypertensive rat (SHR), filled bars/circles, *Cfb*^−/−^, open bars/circles. *P<0.05, **P<0.01, ***P<0.005. G:I indicates ratio of area under the curve of plasma glucose concentration to area under the curve of plasma insulin concentration.

To test whether *Cfb* ablation affected glucose homeostasis in SHR, oral glucose tolerance and insulin sensitivity (IVITT [intravenous insulin tolerance test]) were assessed. Fasting plasma glucose concentration in *Cfb*^−/−^ was significantly lower than SHR (Figure 1A; SHR, 4.62±0.10 versus *Cfb*^−/−^, 4.25±0.09; *P*=0.013). Throughout the oral glucose tolerance, blood glucose remained lower, and area under the glucose curve was significantly reduced in *Cfb*^−/−^ compared with SHR; insulin concentrations were similar in both groups (Figure 1A and 1B). Together with the G:I ratio (ratio of area under the curve of plasma glucose concentration to area under the curve of plasma insulin concentration; Figure 1C), this indicated an improvement in insulin sensitivity, further demonstrated in IVITTs by a significant 48% increase in insulin-stimulated glucose disposal (K_ITT_) in *Cfb*^−/−^ compared with SHR (Figure 1D).

### Adipose Tissue Function

**Figure 2.**
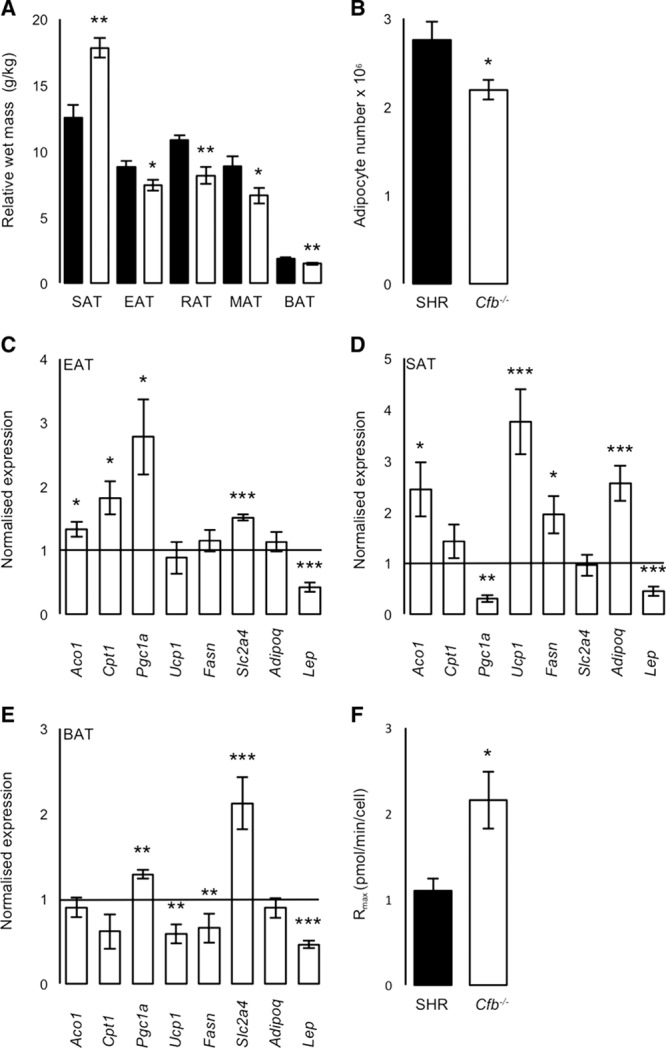
Adipose tissue and adipocyte morphometry, gene expression, and respiratory capacity. **A**, Adipose tissue wet masses, including subcutaneous (SAT), epididymal (EAT), retroperitoneal (RAT), mesenteric (MAT), and brown (BAT; n=6 per group). **B**, Epididymal mean cell number (n=6 per group). **C**, EAT, (**D**) SAT, (**E**) BAT gene expression levels in *Cfb*^−/−^, normalized to *Actb* (n=5 per group). **F**, Maximal respiratory rates in primary epididymal adipocytes. Spontaneously hypertensive rat (SHR), filled bars, *Cfb*^-/-^, and open bars. *Aco1* indicates aconitase 1; *Adipoq*, adiponectin; *Cpt1*, carnitine palmitoyltransferase I; *Fasn*, fatty acid synthetase; normalized expression, gene of interest normalized to β-actin; *Lep*, leptin; *Pgc1a*, peroxisome proliferator-activated receptor gamma coactivator 1 alpha *Slc2a4*, solute carrier family 2 member 4; and *Ucp1*, uncoupling protein 1. *P<0.05, **P<0.01, ***P<0.005.

To determine whether *Cfb* affects adipose function, as suggested by our previous quantitative trait transcript analysis and metabolic phenotyping, we measured adipose tissue depots masses. Relative wet masses of visceral (epididymal adipose tissue [EAT]; mesenteric adipose tissue [MAT]; and retroperitoneal adipose tissue) and brown fat (brown adipose tissue [BAT]) were significantly reduced in *Cfb*^−/−^ rats compared with SHR, despite similar total body mass (269±20 versus 265±31 g; *P*>0.05; Figure 2A); however, *Cfb*^−/−^ had significantly more relative subcutaneous fat (SAT; Figure 2A). Overall, total fat mass was similar (SHR, 42.9±1.4 versus *Cfb*^−/−^, 42.8±1.4 g/kg; *P*>0.05). Stereological analysis of EAT showed that *Cfb*^−/−^ had significantly fewer, similar-sized adipocytes than SHR (SHR, 4.06±0.21 versus *Cfb*^−/−^, 4.13±0.32×10^5^ μm^3^ *P*>0.05; Figure 2B). Further, serum analysis of circulating lipids and adipokines demonstrated significant decreases in levels of cholesterol, triglycerides, and high molecular-weight adiponectin (−Δ48%), in *Cfb*^−/−^ compared with SHR; however, circulating total adiponectin and leptin were similar (Table S4).

Given the varied metabolic contributions of different fat depots found in the *Cfb*^−/−^ rat, we analyzed transcript abundance for markers of oxidation (*Cpt1*and *Aco1*), beigeing (*Ucp1* and *Pgc1a*), insulin sensitivity (*Slc2a4*), lipid metabolism (fatty acid synthase [*Fasn*]), and adipokines (*Adipoq* and *Lep*). In EAT, *Pgc1a*, *Cpt1*, *Aco1*, and *Slc2a4* were significantly increased in *Cfb*^−/−^ compared with SHR (Figure 2C). In SAT, *Aco1*, *Ucp1*, *Fasn*, and *Adipoq* were significantly elevated, whereas *Pgc1a* was reduced, in *Cfb*^−/−^ compared with SHR (Figure 2D). In BAT, *Pgc1a* and *Slc2a4* were significantly increased in *Cfb*^−/−^ compared with SHR, whereas *Ucp1* and *Fasn* were significantly decreased (Figure 2E). *Lep* was significantly reduced in all *Cfb*^−/−^ depots compared with SHR (Figure 2C through 2E).

To determine whether transcript changes were associated with altered adipose tissue respiration, we analyzed epididymal adipocyte metabolic rate. Maximal and basal respiratory rates were significantly greater in *Cfb*^−/−^ than in SHR, +Δ1.64, and +Δ1.96-fold, respectively (Figure 2; Figure S2A). Further, reserve capacity and leak respiration were both significantly increased (Figure S2B and S2C). However, ATP-linked respiration and ATP-generation efficiency were similar (Figure S2D through S2E). CoxIV protein abundance—a mitochondrial marker—was similar in both *Cfb*^−/−^ and SHR (Figure S2F).

There were no differences in body temperature or activity associated with *Cfb* deletion (Figure S3A and S3B).

### Cardiovascular Analyses

**Figure 3.**
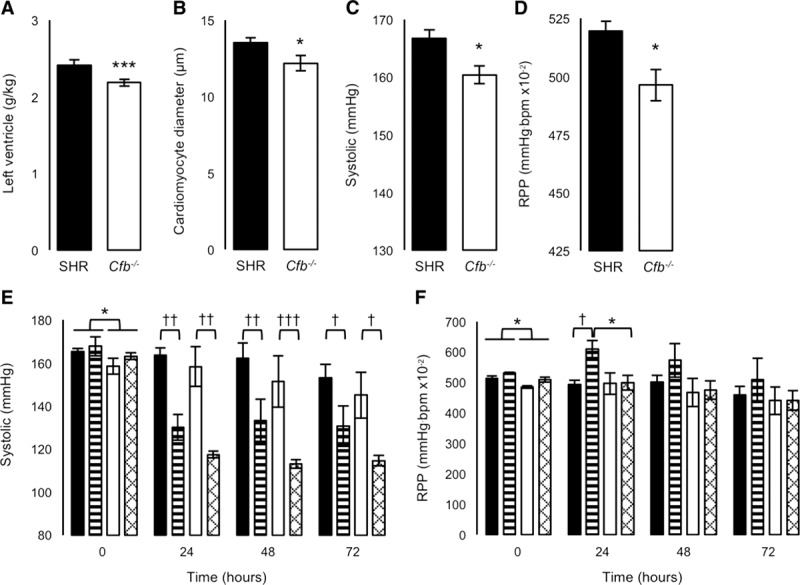
Left ventricle morphometry, blood pressure, and rate pressure product before and after 72-h infusion of isoproterenol or saline. **A**, Left ventricle wet mass and (**B**) mean left ventricular cardiomyocyte diameter. **C**, baseline mean systolic blood pressure and (**D**) rate pressure product recorded telemetrically. **E**, Mean systolic blood pressure and (**F**) rate pressure product recorded telemetrically during infusion of isoproterenol or saline. Black-filled bars, spontaneously hypertensive rat (SHR), saline-treated; stripe-filled bars, SHR, isoproterenol-treated; white-filled bars, *Cfb*^-/-^, saline-treated; hatch-filled bars, *Cfb*^-/-^, isoproterenol-treated. Differences in genotype *P<0.05, ***P<0.0005 or treatment †*P*<0.05, ††*P*<0.005, †††*P*<0.0005.

*Cfb* deletion reduced relative LV mass and cardiomyocyte diameter by 10% compared with SHR; however, relative heart weight was similar between genotypes (Figure 3A and 3B; Figure S4A and S4B). Telemetrically measured systolic and diastolic blood pressures were significantly lower (−Δ7 mm Hg) in *Cfb*^−/−^ than in SHR, and although heart rate was similar, rate pressure product was significantly reduced (Figure 3C and 3D; Figure S4C through S4F). Serum aldosterone and transcripts for renal renin and hepatic angiotensinogen were all significantly reduced in *Cfb*^−/−^ rats (Table S4, Figure S5A and S5B).

Early structural and functional changes in the heart were investigated using echocardiography. We confirmed that relative LV mass was significantly reduced in *Cfb*^−/−^ compared with SHR; however, at this stage, LV wall thickness was not significantly different (Table S5). Functionally fractional shortening and ejection fraction were significantly increased in *Cfb*^−/−^ LV compared with SHR (Table S5). Given the similar heart rate and stroke volume, cardiac output was not significantly different (Table S5).

**Figure 4.**
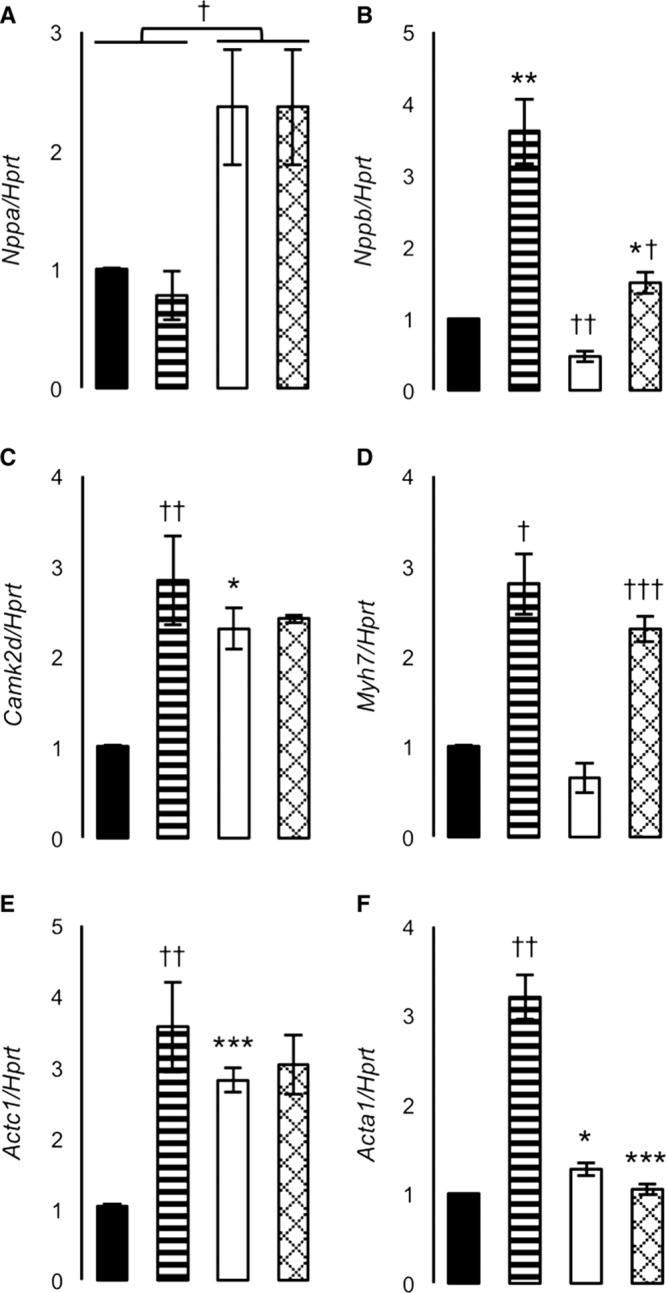
Gene expression levels in left ventricles after 72-h isoproterenol or saline treatment. **A**, *Nppa*, natriuretic peptide a, (**B**) *Nppb*, brain natriuretic peptide, (**C**) *Camk2d*, calcium/calmodulin dependent protein kinase II delta *Myh6*, (**D**) *Myh7*, myosin heavy polypeptide 7, (**E**) *Actc1*, α-cardiac actin, (**F**) *Acta1*, α-skeletal actin. Black-filled bars, SHR, saline-treated; stripe-filled bars, spontaneously hypertensive rat (SHR), isoproterenol-treated; white-filled bars, *Cfb*^-/-^, saline-treated; hatch-filled bars, *Cfb*^-/-^, isoproterenol-treated. Differences in genotype *P<0.05, **P<0.005, ***P<0.0005 or treatment †*P*<0.05, ††*P*<0.005, †††*P*<0.0005.

An acute hypertrophic challenge designed to investigate whether *Cfb* deletion conferred protection from cardiac stress, independent of blood pressure, showed that the rate pressure product was significantly reduced in *Cfb*^−/−^ hearts in the 24 hours after isoproterenol treatment (Figure 3E and 3F; Figure S6A). Isoproterenol increased relative heart and LV mass similarly (Figure S6B and S6C). Transcripts related to cardiac hypertrophy were investigated in LV from isoproterenol and saline-treated rats. In saline-treated *Cfb*^−/−^ rats, *Nppa*, *Actc1*, and *Camk2d* were significantly increased compared with SHR (Figure 4A, 4C, and 4E); whereas *Nppb* was significantly decreased (Figure 4B). In isoproterenol-treated rats, *Nppb* increased marginally in *Cfb*^−/−^ rats compared with SHR (Figure 4B). *Acta1* in isoproterenol-treated *Cfb*^−/−^ rats was similar to both saline-treatment groups (Figure 4F). The ratio of *Actc1*:*Acta1* was significantly greater in *Cfb*^−/−^ compared with SHR, in saline-treated (317±43 versus 138±18; *P*=0.05) and isoproterenol-treated rats (256±37 versus 53±9; *P*<0.005). *Myh6* and *Myh7* expression was similar between genotypes (Figure 4D; Figure S7).

### Serum Markers of Inflammation

Given the function of *Cfb* in inflammatory responses, we determined the effect of *Cfb*^−/−^ on Th-1 mediated inflammation by quantifying serum concentrations of cytokines (Il-2, Il-6, Il-10, granulocyte macrophage colony stimulating factor, Ifn-γ, and Tnfα). We found significant decreases in serum concentrations of Il-10 and Ifn-γ in *Cfb*^−/−^ rats compared with SHR. In addition, whereas Il-6 and Tnfα were detected in SHR, the cytokines were undetectable in sera from *Cfb*^−/−^ rats. Granulocyte macrophage colony stimulating factor was similar in both groups, and in neither group was Il-2 detected (Table S4).

### Analysis of GWAS Hits and *cis*-Expression QTLs at the Human *CFB* Locus

**Figure 5.**
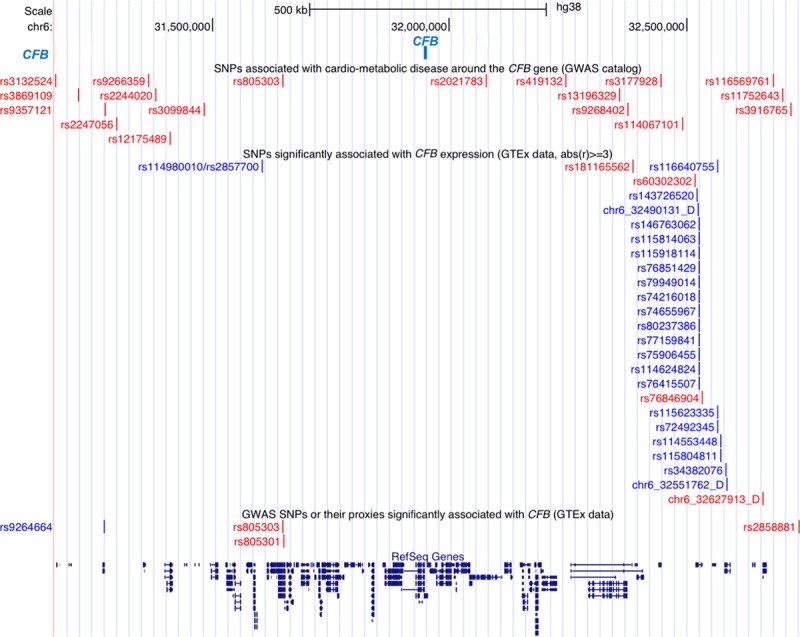
Cardiometabolic genome-wide association study (GWAS) hits and *cis*-eQTLs (quantitative trait loci) located in the human the complement factor B (*CFB*) locus. Eighteen relevant cardiometabolic single-nucleotide polymorphisms (SNPs) located <1 Mb from the boundaries of the human *CFB* gene (upper; red). Twenty-six SNPs were retrieved from the GTEx Portal that were found to be significantly associated with *CFB* expression (*P*<0.05), blue SNPs are associated with a significant negative effect, whereas red SNPs are associated with a significant positive effect. Four SNPs (with 1 overlapping) were determined to be correlated to both *CFB* expression, as well as being GWAS hits for relevant cardiometabolic traits (lower; red/blue). See Table S8 for a list of genes located in the *CFB* locus.

To determine whether genetic variants near *CFB* are associated with metabolic and cardiovascular disorders relevant to MetS (Table S3), we mined the NHGRI GWAS catalog (National Human Genome Research Institute) and located 18 single-nucleotide polymorphisms (SNPs) associated with cardiometabolic traits ≤1 Mb from *CFB* (Figure 5; Table S6). Six SNPs were found to be associated with type 2 diabetes mellitus, MetS, or visceral fat. Six further SNPs were related to circulating lipids. The remaining SNPs were associated with coronary heart disease and hypertension (Table S6).

We also investigated whether variants at the *CFB* locus are associated with *CFB* expression by mining GTEx datasets (the Genotype-Tissue Expression project) for *CFB cis*-expression quantitative trait loci (QTLs). Fifty-three SNPs were associated with *CFB* expression in 4 tissues (Figure 5; Table S7). One SNP, rs76846904, close to the *HLA-DRB5* gene, is highly correlated with *CFB* gene expression in subcutaneous adipose tissue (effect size, 0.78; *P*=0.000015) and within 100 kb of GWAS hits for visceral adiposity, serum cholesterol, and coronary heart disease.

The influence of the 18 GWAS SNPs, or any of their proxies (a total of 280 SNPs), on gene expression across 9 tissues was investigated using the GTEx Portal. Four SNPs were significantly associated (false discovery rate<0.05) with *CFB* expression in tissues of interest (Figure 5; Tables S6 and S7). Two SNPs, correlating with *CFB* expression in “adipose subcutaneous” and “artery aorta”, respectively, are proxies for rs13196329 and rs2247056, which are associated with visceral fat and triglycerides in the GWAS catalog (Table, Figure 5). Two further SNPs were significantly associated with increased *CFB* expression in “heart LV” and correspond to the same SNP (rs805303) that is associated with increased systolic and diastolic blood pressure and hypertension in the GWAS catalog (Table; Figure 5).

**Table.**
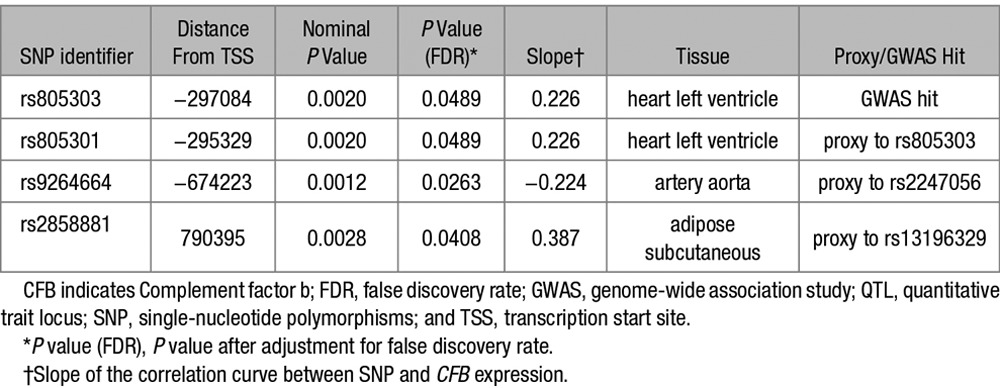
cis-eQTL SNPs Significantly Correlated With CFB Gene Expression and GWAS Hits

## Discussion

We tested the hypothesis that *Cfb* is necessary for the full expression of cardiometabolic pathophysiological traits in the SHR model of MetS. Through ZFN-mediated gene knockout, we showed that the *Cfb*-deficient (*Cfb*^−/−^) SHR has improved glucose tolerance and insulin sensitivity, along with favorable adipose tissue distribution, adipose oxidative capacity, and reduced circulating lipids and proinflammatory cytokines compared with parental SHR. Further, *Cfb*^−/−^ rats had reduced blood pressure that was associated with increased ejection fraction and fractional shortening and reduced LV mass. The human *CFB* locus—a gene-rich region within the major histocompatibility complex—contains several GWAS hits for cardiometabolic traits, including coronary heart disease, blood pressure, MetS, type 2 diabetes mellitus, serum lipids, and visceral fat. These colocalize with *cis*-expression QTLs associated with expression of *CFB* in subcutaneous adipose tissue and other tissues, indicating that variation in *CFB* expression may underlie, in part, the GWAS hits at this locus.

Glucose intolerance, insulin resistance, visceral adiposity, and dyslipidemia are the key metabolic features of MetS that increase the risk of type 2 diabetes mellitus.^23^ In our study, *Cfb*^−/−^ rats had reduced visceral but increased subcutaneous fat. To investigate potential molecular changes associated with favorably altered fat distribution and ameliorated glucose homeostasis in *Cfb*^−/−^ rats, we investigated transcripts central to adipose tissue metabolism. Reduced EAT mass in *Cfb*^−/−^ rats was because of reduced adipocyte number rather than altered adipocyte volume. *Pgc1a, Cpt1*, and *Aco1* were upregulated in *Cfb*^−/−^ rats, suggestive of increased adipocyte oxidative phosphorylation, which we confirmed by Seahorse analysis. *Cfb*^−/−^ rats exhibited a marked increase in basal and maximal respiration and had a 2-fold increased reserve respiratory capacity. Taken together with the reduction in adipocyte number, the data suggest that the elevation of mitochondrial respiratory capacity may provide an adipose tissue-intrinsic mechanism for reduced fat accumulation in *Cfb*^−/−^ EAT. In SAT, increased mass in *Cfb*^−/−^ rats was associated with increased *Fasn* and reduced *Pgc1a* expression, consistent with the function of *Fasn* as an insulin-sensitive fatty acid synthase, the role of Pgc1a in stimulating fatty acid oxidation, and the known upregulation of *FASN* in human obesity and type 2 diabetes mellitus.^24^ These changes seemed to override the increases in *Aco1* and *Ucp1* expression observed in *Cfb*^−/−^ rats, which would be expected to reduce adipocyte mass through increased trichloroacetic acid cycle activity and thermogenesis. The redistribution of visceral to subcutaneous fat marked changes in gene expression, and adipose respiratory capacity are likely to be the key to improvements in whole-body glucose homeostasis and metabolic function in *Cfb*^−/−^ rats. Reduced BAT mass in *Cfb*^−/−^ rats was associated with increased *Pgc1a* and *Slc2a4* and decreased *Ucp1* and *FASN* expression. This fat reduction may be consistent with increased *Pgc1a* driving lipolysis although inhibiting fatty acid synthesis; however, further experiments in *Cfb*^−/−^ rats will be required to understand the BAT energy-substrate balance resulting from *Cfb* deficiency.

To further investigate altered adipose function in the *Cfb*^−/−^ rat, we quantified *Lep* and *Adipoq* transcripts in EAT, SAT, and BAT. Although adipose *Lep* expression was reduced, circulating leptin was comparable in *Cfb*^−/−^ and SHR. Although incompletely explained here, this could be accounted for by differences in post-translational processing and release, or peripheral metabolism, of leptin. Despite increased *Adipoq* expression in SAT alone, circulating high molecular-weight adiponectin was reduced in *Cfb*^−/−^ rats. Conversely, high molecular-weight adiponectin in humans is lower in obese, insulin-resistant compared with lean, insulin-sensitive individuals.^25^ However, adiponectin deficiency in mice has been shown to have no effect on glucose homeostasis on a normal diet.^26,27^ Further, infusion of adiponectin in high-fat fed SHRs only marginally reduced insulin levels without affecting energy expenditure or hypertension.^28^ Taken together with the observed metabolic improvements, this suggests other mechanisms, besides adiponectin, drive insulin sensitization in the *Cfb*^−/−^ rat.

We also tested the hypothesis that deletion of *Cfb* in SHR would affect the expression of SHR cardiovascular phenotypes. In this study, we showed that *Cfb*^−/−^ rats had reduced systolic and diastolic blood pressure, reduced LV mass and cardiomyocyte diameter, and an abrogated isoproterenol-induced increase in rate pressure product. These alterations represent a marked amelioration in several of the key cardiovascular features of MetS manifested in SHR.

The reduction in blood pressure was associated with reductions in renin–angiotensin system components, suggesting that *Cfb* may have a direct effect, yet unexplained, on this system, mediating blood pressure and subsequently LV mass. Although *Cfb* deletion leads to lower blood pressure in SHR, our experiments do not distinguish whether *Cfb* is responsible for increasing above or maintaining basal blood pressure. Further detailed experiments are required to distinguish these 2 possible mechanisms.

To gain further insight into the molecular changes caused by *Cfb* deficiency in the heart, we investigated the effect of *Cfb* deletion on cardiomyogenic genes (ie, *Nppa*, *Nppb*, *Myh6*, *Myh7*, *Acta1*, and *Camk2d*), which are activated in response to stress.^29^ Our study showed that despite reduced LV mass, *Camk2d* expression was significantly increased in saline-treated *Cfb*^−/−^. CaMKII (calcium/calmodulin-dependent protein kinase type 2) is proposed to regulate inflammation (*Cfb*, *Tnfa*, and *Il-6*) and cardiomyogenesis in response to hypertension-related pressure overload, β-adrenergic agonists, or myocardial infarction-induced cell injury.^30^ Thus, *Cfb* may contribute to both cardiac inflammation and hypertrophy in response to stress, possibly through regulation of cardiomyogenic gene expression. For example, we showed complete or near complete abrogation in *Cfb*^−/−^ rats of the isoproterenol-stimulated increase in *Acta1* and *Nppb* expression seen in SHR. Further, *Nppa* expression was increased in both saline- and isoproterenol-treated *Cfb*^−/−^. Therefore, independent of blood pressure, the lack of compensatory *Acta1* upregulation and the favourable *Actc1*:*Acta1* ratio^31^ indicate that the *Cfb*^−/−^ LV may be partially protected from compensatory cytoskeletal changes associated with LV dysfunction. Equally, abrogation of *Nppb* expression in the presence of isoproterenol indicates that the *Cfb*^−/−^ LV is partly protected from stress. Further, upregulation of *Nppa* in *Cfb*^−/−^ rats may, in part, contribute to the observed reduction in cardiomyocyte diameter and LV mass. Taken together, in *Cfb*^−/−^ rats, upregulation of *Nppa* and abrogation of *Acta1* expression in the presence of isoproterenol may indicate a blood pressure-independent mechanism for preserving LV function.

In addition to glucose metabolism and hypertension, we assessed the concentration of circulating lipids and Th-1 cytokines and showed reduced cholesterol and triglycerides, as well as reduced proinflammatory cytokines in *Cfb*^−/−^ rats. Some of the metabolic and immune parameters that we measured here have also been measured in a *Cfb*^−/−^ mouse, although no cardiovascular measurements have been reported. Like the *Cfb*^−/−^ rat, the *Cfb*^−/−^ mouse lacks AP activity and has reduced Tnfα, Il-6, and Ifn-γ.^32,33^ Although having some immune similarities to the *Cfb*^−/−^ rat, *Cfb*^−/−^ mice compared with WT mice are more glucose intolerant and have higher circulating triglycerides.^34^ The differences between these 2 models could be because of several reasons, including genetic background affecting metabolism differently, the use of high-fat diet in the mouse studies to elicit a phenotype, and the presence of 2 protein-coding *Cfb* transcripts in the mouse, whereas rats and humans have only one. On a high-fat diet, *Ldlr*^−/−^*/Cfb*^−/−^ mice showed protection against atherosclerosis,^35^ which is distinct from the amelioration in metabolic and cardiovascular phenotypes that we observed here. However, the 2 studies combined strongly encourage further investigation of Cfb as a target for protection from the development of cardiovascular disease.

Rat *Cfb* resides in chromosome 20p12, a region previously found to be important in the regulation of blood pressure, glucose homeostasis, and adiposity in SHR.^18–21^ We propose that *Cfb*, at least in SHR, plays a major part in the development of key features of MetS that are linked to 20p12. However, given that the SHR.1N congenic that covers 20p12 has a reduction of 20 mm Hg, other genes in the region may also contribute.^19^

The location of human *CFB* and the syntenic region to the rat gene is on human 6p21.33.^18^ We located 18 SNPs with genome-wide significant associations to cardiometabolic traits ≤1 Mb from *CFB*. Several GWAS hits in the region were associated with type 2 diabetes mellitus and components of MetS. Two SNPs, rs13196329 and rs2247056, were correlated with visceral fat, triglycerides, and *CFB* expression. Further, 1 SNP, rs805303, was significantly positively correlated with systolic and diastolic blood pressure, and hypertension, as well as with increased *CFB* expression. These results suggest that *CFB* expression associated with these SNPs may be causally linked to accumulation of visceral fat, circulating lipids, and development of hypertension in humans.

In addition to altering complement activity, *Cfb* ablation reduced proinflammatory cytokines Ifn-γ, Il-6, and Tnfα whose elevated levels are associated with hypertension, obesity, and insulin resistance.^36,37^ Further, chronic low-grade inflammation and overactivation of the innate immune system are now recognized causes of type 2 diabetes mellitus,^4,5^ with clinical trials for therapeutic targets against inflammatory pathways for the treatment of diabetes mellitus and cardiovascular disease currently underway.^38^

Compounds that target CFB already exist, and taken together with the findings in our study, suggest that CFB has significant potential as a novel target for treatment of metabolic disease^39,40^

This is the first study to report the widespread amelioration of metabolic and cardiovascular phenotypes through deletion of an alternative complement pathway gene in a model of MetS. *Cfb* deletion improves glucose homeostasis, adipose distribution and function, lowers blood pressure and reduces cardiac hypertrophy, protecting against LV stress. Together with our analysis of the human *CFB* region for cardiometabolic traits, we conclude that *CFB* expression and function may directly or indirectly regulate multiple metabolic and cardiovascular processes in health and disease in the rat and in humans.

## Perspectives

CFB is elevated in human cohorts with type 2 diabetes mellitus and cardiovascular disease, although a causal relationship has yet to be established. We identified alterations in *Cfb* expression as a possible cause of hypertension and insulin resistance in the SHR. *Cfb* knockout rats have improved glucose homeostasis linked to favorable alterations in adipose tissue distribution and function and reduced blood pressure and LV mass suggesting new adipose tissue-intrinsic and blood pressure-independent mechanisms for SHR insulin resistance and cardiac hypertrophy. SNPs in human *CFB* are associated both with hypertension and visceral adiposity and with *CFB* gene expression, suggesting that genetic variation in *CFB* may, in part, explain the genetic associations at the human *CFB* locus. Further studies are required to establish whether overexpression of adipose tissue *Cfb* alone is the prime determinant of MetS traits. Clinical trials are presently being undertaken to test the therapeutic effects of CFB inhibitors and to investigate AP components as causal factors in human diseases related to overactivity of the innate immune system. Given the findings in this study, CFB may also be a valid therapeutic target to treat or prevent progression of human MetS.

## Acknowledgments

Ultrasound imaging was performed by A.T. of Edinburgh Preclinical Imaging, University of Edinburgh. Histological processing and flow cytometry was carried out by the Shared University Research Facilities and Queen's Medical Research Institute Flow Cytometry and Cell Sorting Facility, Edinburgh. Whole genome sequencing was performed at Edinburgh Genomics Clinical Genomics, Edinburgh, United Kingdom. T.J.A. has received speaker honoraria from Illumina, Inc, and consultancy fees from AstraZeneca.

## Sources of Funding

P.M.C., M.B., N.A., A.G.D., and J.M. are funded by an Advanced Grant ERC-2010-AdG_20100317 (ELABORATE; elucidation of the molecular and functional basis of disease phenotypes in the rat model) from the European Research Council awarded to T.J.A. R.N.C. and N.M.M. are funded by a Wellcome Trust New Investigator grant 100981/Z/13/Z awarded to N.M.M. S.M.P. and L.H.J.-J. are funded by Medical Research Council grants MR/N005902/1 and MR/M011542/1, respectively. Radiotelemetry equipment was funded by a Wellcome Trust Institutional Strategic Support Fund (ISSF2) award J22737 and the British Heart Foundation Centre of Research Excellence, University of Edinburgh.

## Disclosures

T.J.A. has received speaker honoraria from and has research collaborations with Illumina and has received consultancy fees from AstraZeneca. The other authors report no conflicts.

## Supplementary Material

## Novelty and Significance

### What Is New?

- Cfb (complement factor B)—an innate immune component—is a determinant of adipose tissue distribution, glucose homeostasis, blood pressure, and left ventricle mass in the spontaneously hypertensive rat.

### What Is Relevant?

- Cfb, directly or indirectly, drives novel adipose tissue-intrinsic and blood pressure-independent mechanisms for insulin resistance, hypertension, and cardiac hypertrophy in the spontaneously hypertensive rat. Single-nucleotide polymorphisms associated with cardiometabolic traits and *CFB* gene expression, suggest variation in *CFB* may, in part, underlie these traits in humans.

### Summary

Metabolic and cardiovascular components of MetS are improved by ablation of the *Cfb* gene in the spontaneously hypertensive rat. At the human *CFB* locus, 3 single-nucleotide polymorphisms are significantly associated with visceral adiposity, hypertension, and *CFB* gene expression.
