# Supplementary material for: Complement Factor B Is a Determinant of Both Metabolic and Cardiovascular Features of Metabolic Syndrome
Source: Hypertension. 2017 Aug 9;70(3):624–33. doi: 10.1161/HYPERTENSIONAHA.117.09242 (PMC5548512; doi:10.1161/HYPERTENSIONAHA.117.09242)
Supplement: Supplementary file 1 [file hyp-70-624-s001.pdf]

COMPLEMENT FACTOR B IS A DETERMINANT OF BOTH METABOLIC AND CARDIOVASCULAR FEATURES OF METABOLIC SYNDROME

Short title: complement factor b knockout rat

Philip M. Coan [1,2], Marjorie Barrier* [1,2], Neza Alfazema* [1,2], Roderick N. Carter [2], Sophie Marion de Procé [1], Xaquin C. Dopico [1,6], Ana Garcia Diaz [3], Adrian Thomson [2], Lucy H. Jackson-Jones[2], Ben Moyon [4], Zoe Webster [4], David Ross [1], Julie Moss [1], Mark J. Arends [5], Nicholas M. Morton [2], Timothy J. Aitman [1,2,3].

[1] Centre for Genomic and Experimental Medicine, MRC Institute for Genetics and Molecular Medicine, Edinburgh, EH4 2XU, UK.
[2] University/British Heart Foundation Centre for Cardiovascular Science, Queen's Medical Research Institute, University of Edinburgh, EH16 4TJ, UK.
[3] Department of Medicine, Imperial College London, London, SW7 2AZ, UK.
[4] Embryonic Stem Cell and Transgenics Facility, MRC Clinical Sciences Centre, Imperial College London, London W12 0NN, UK.
[5] Division of Pathology & Centre for Comparative Pathology, Edinburgh Cancer Research UK Cancer Centre, Institute of Genetics & Molecular Medicine, Edinburgh, EH4 2XR, UK.
[6] Royal (Dick) School of Veterinary Studies, University of Edinburgh, EH25 9RG, UK.

*Equal contribution

Corresponding author:

Dr Philip M. Coan, Centre for Genomic and Experimental Medicine, MRC Institute for Genetics and Molecular Medicine, Edinburgh, EH4 2XU, UK.
+44 (0) 131 242 6690
p.m.coan.02@cantab.net

Supplemental Methods

Rats

Cfb$^{-/-}$ rats were generated on an SHR/NCrl background (Charles River, Margate, UK), by microinjecting ZFN mRNA (Sigma), targeted to exon 6 of *Cfb* (target sequence: CCCCTCGGGCTCCATGaatatcTACATGGTGCTGGATG), into one-cell stage SHR/NCrl embryos that were implanted into pseudopregnant rats. Heterozygous progeny, from a founder harboring a 19 bp deletion in *Cfb*, were intercrossed to generate homozygous knockout rats. A search for off-target events was conducted by whole genome sequencing and analysed as described previously, confirmed the 19 bp deletion [1, 2]. Six additional putative variants, analysed by Sanger Sequencing, were determined to be false positives (Table S1). Rats were housed in open cages with free access to food and water. All procedures were carried out in accordance with UK Home Office regulations.

Serum analysis

Following an overnight fast, serum was extracted from whole blood exsanguinated under terminal isofluorane anaesthesia (n = 6 per group). Serum lipids were analysed by the Veterinary Pathology Laboratory, Edinburgh. In-house ELISAs were used to determine: serum Alternative complement (AP) activity (Hycult Biotech), leptin and total adiponectin (Merck Millipore), and high-molecular-weight (HMW) adiponectin and aldosterone (AMS Biotech). Serum Th1 cytokine concentrations were quantified using the LEGENDplex Rat Th1 Panel (6-plex) kit (BioLegend) and BD Accuri™ C6 Flow Cytometer (BD Biosciences). Those cytokines reported undetectable, were below the sensitivity of the assay.

Adipocyte morphometry

Epididymal fat pads were weighed, cut into five equal pieces, and processed for paraffin wax embedding (n = 6 rats per group). A random image was taken from one 4 µm thick H&E stained section per piece at 20x magnification to estimate mean adipocyte volume [3]: a line grid was superimposed on to each image and point sampled intercept lengths (PSI) measured between two points on the cell membrane. One hundred PSI were measured per pad and adjusted for shrinkage [4]. Fat pad weight was converted to volume according to Farvid *et al* [5], which was then divided by mean adipocyte volume to estimate volume-weighted adipocyte number.

Glucose homeostasis

Oral glucose tolerance (OGTT) (n = 10 per group) and intravenous insulin tolerance tests (IVITT) (n = 7 per group) were performed as described [6, 7]. Glucose clearance (K_{ITT}) was calculated as described [8].

Adipocyte metabolic rate

Isolated primary rat adipocytes (n = 6 rats per group) in Kreb's buffer (118 mM NaCl, 1.2 mM $MgSO_4$, 15 mM $NaPO_4$, 1.265 mM $CaCl_2$, 5.56 mM Glucose, 1% BSA) were adhered to Matrigel (Corning) coated Seahorse plates (Agilent), washed with XF-DMEM (Agilent, supplemented with 1 mM Pyruvate and 10 mM Glucose, pH 7.4), and incubated (37°C, without CO_2, 15 min). A mitochondrial stress test was performed as described previously [9] in an XFe24 Seahorse Bioanalyser (Agilent) and oxygen consumption rate data calculated according to the manufacturer's instructions (Agilent Technologies LDA UK, Cheshire, UK).

Telemetry
Blood pressure transmitters were implanted, using isofluorane anaesthesia, according to manufacturer's instructions (HD-S10, Data Sciences International). Following surgical recovery (>7 days), blood pressure, temperature and activity were recorded for 72 h (5 min/h) (n = 8-9 per group), before subcutaneous implantation of osmotic pumps, under brief isoflurane anaesthesia, (1003D, Azlet) containing either isoproterenol (1.2 mg/kg/h) or saline (n = 4-5 per group), and further data collected for 72 h.

Echocardiography
In vivo ultrasound echocardiography was performed by using a Vevo 770 ultrasound biomicroscope (Visualsonics) with a RMV710B 25 MHz center frequency transducer in 7 week-old male rats. Briefly, isoflurane anesthetized rats were placed on a thermostatically controlled ECG monitoring table and maintained at 37°C. Parasternal long axis (PLAX) ECG-Gated Kilohertz Visualisation (EKV) B mode and M-mode views of the left ventricle (LV) were acquired. LV end-systolic and end-diastolic areas were measured by tracing the endocardial border using Vevo Analysis Software (Visualsonics) in order to calculate ejection fraction (EF) from the PLAX EKV B mode view and fractional shortening from the M-mode view.

Cardiomyocyte diameter
Left ventricle mean cardiomyocyte diameter was determined as described previously [10] using images taken by QImaging Micropublisher 3.3RTV camera (QImaging) attached to an Olympus BX51 microscope (Olympus) and measured using the STEPanizer program (n = 8 per group).

Gene expression
RNA was extracted from fat depots (subcutaneous (SAT), epididymal (EAT) and brown fat (BAT)) (n = 6 per group) and left ventricle (LV) (n = 4-5 per group) for qPCR, as described previously [8]. Primer sequences are listed in Table S2. *Actb* was used as a reference gene for adipose transcripts and LV transcripts. LV transcripts from telemetric studies were normalised to *Hprt*, due to effects of isoproterenol on *Actb* expression. Ct values were compared using the $2^{-\Delta\Delta Ct}$ method.

***In silico* analysis of the CFB locus**
Single-nucleotide polymorphisms (SNPs) associated with cardio-metabolic traits related to type 2 diabetes and MetS residing ≤1 Mb from human *CFB* (Table S3) were identified by mining the NHGRI GWAS catalog [11]. Proxy SNPs, based on linkage disequilibrium were determined using SNAP (https://archive.broadinstitute.org/mpg/snap/ldsearchpw.php) with the 1000 genomes Pilot 1 and HapMap (release 21 and 22) databases using default parameters (0.8 r^2 threshold, 500nt distance). GWAS and proxy SNP locations (280 in total) were converted to hg19 coordinates using dbSNP [12] and the UCSC Liftover tool [13]. Associations between SNPs and *cis*-regulated expression quantitative trait loci (*cis*-eQTLs) ≤1 Mb from *CFB* transcription start site (TSS) were determined from tissue data files (adipose subcutaneous, artery tibial, adipose visceral omentum, artery aorta, heart atrial appendage, heart left ventricle, pancreas, artery coronary, and liver) for SNP-gene association pairs downloaded from the GTex portal

(http://www.gtexportal.org/home/). False discovery rate (FDR) was determined in R according to the Benjamini-Hochberg approach (https://www.r-project.org).

References

1. Atanur SS, Diaz AG, Maratou K*, et al.* Genome sequencing reveals loci under artificial selection that underlie disease phenotypes in the laboratory rat. *Cell*. 2013;154:691-703.
2. Van der Auwera GA, Carneiro MO, Hartl C*, et al.* From fastq data to high confidence variant calls: The genome analysis toolkit best practices pipeline. *Curr Protoc Bioinformatics*. 2013;43:11 10 11-33.
3. Tschanz SA, Burri PH, Weibel ER. A simple tool for stereological assessment of digital images: The stepanizer. *J Microsc*. 2011;243:47-59
4. Gundersen HJ, Jensen EB. Stereological estimation of the volume-weighted mean volume of arbitrary particles observed on random sections. *J Microsc*. 1985;138:127-142.
5. Farvid MS, Ng TW, Chan DC, Barrett PH, Watts GF. Association of adiponectin and resistin with adipose tissue compartments, insulin resistance and dyslipidaemia. *Diabetes Obes Metab*. 2005;7:406-413
6. Pravenec M, Landa V, Zidek V, Musilova A, Kazdova L, Qi N, Wang J, St Lezin E, Kurtz TW. Transgenic expression of cd36 in the spontaneously hypertensive rat is associated with amelioration of metabolic disturbances but has no effect on hypertension. *Physiol Res*. 2003;52:681-688.
7. Conde SV, Nunes da Silva T, Gonzalez C, Mota Carmo M, Monteiro EC, Guarino MP. Chronic caffeine intake decreases circulating catecholamines and prevents diet-induced insulin resistance and hypertension in rats. *Br J Nutr*. 2012;107:86-95.
8. Coan PM, Hummel O, Diaz AI, Barrier M, Alfazema N, Norsworthy PJ, Pravenec M, Petretto E, Huebner N, Aitman TJ. Genetic, physiological and comparative genomic studies of hypertension and insulin resistance in the spontaneously hypertensive rat. *Dis Model Mech*. 2017:10.1242/dmm.026716.
9. Bugge A, Dib L, Collins S. Measuring respiratory activity of adipocytes and adipose tissues in real time. *Methods Enzymol*. 2014;538:233-247
10. Zhao XY, Li L, Zhang JY, Liu GQ, Chen YL, Yang PL, Liu RY. Atorvastatin prevents left ventricular remodeling in spontaneously hypertensive rats. *Int Heart J*. 2010;51:426-431.
11. Welter D, MacArthur J, Morales J, Burdett T, Hall P, Junkins H, Klemm A, Flicek P, Manolio T, Hindorff L, Parkinson H. The nhgri gwas catalog, a curated resource of snp-trait associations. *Nucleic Acids Res*. 2014;42:D1001-D1006.
12. Sherry S, Ward M, Kholodov M, Baker J, Phan L, Smigielski E, Sirotkin K. Dbsnp: The ncbi database of genetic variation. *Nucleic Acids Res*. 2001;29:308-311.
13. Kent W, Sugnet C, Furey T, Roskin K, Pringle T, Zahler A, Haussler D. The human genome browser at ucsc. *Genome Res*. 2002;12:996-1006.

Supplementary Tables

Table S1. Putative ZFN off-target events that were found to be false positives

Gene name	Off-target position	Rnor_6.0	SHR/NCrl (Illumina)	*Cfb*$^{-/-}$ (Illumina)	SHR/NCrl (Sanger)	*Cfb*$^{-/-}$ (Sanger)
Grb2	1:98046688	GCCC	GC/GC	G/GC	GCCC	GCCC
Abhd17c	1:146289241	C	C/C	C/G	C	C
AABR07051532.1	3:16440449-749	C	G/G	G/T	C	C
AABR07065498.1	6:132175624	A	ACCCCC/ ACCCCC	ACCCC/ ACCCCC	A	A
AABR07065768.3	6:140407070	T	T/C	G/C	T	T
Ppidl1	9:121457023-68	C	C/C	C/T	C	C

Table S2. Primer sequences used for quantitative real-time PCR analysis

Gene	Forward	Reverse
Aco1	TCAGATAAAGCTGGACACCGGG	CCTACTGGGCCATCTTTCGGAT
Actb	ATGTACCCAGGCATTGCTGAC	GAGTACTTGCGCTCAGGAGGA
Actc1	CAAAGCACGCCTACAGATCCCA	GAAGACAGCTCTGGGAGCATCA
Adipoq	CTCCACCCAAGGAAACTTGTGC	TTAGGACCAAGAACACCTGCGT
Agt	GCTGGAGCTAAAGGACACACAG	AAAGGGGTGGATGTATACGCGG
Camk2d	AGTGAGGCTGATGCCAGTCATT	CAGGTCCCTGTGAACTATGCCA
Cfb	AGTAGAGATCAAAGGCGGCTCC	TTCGAGTCTGCACAGGGTATGG
Cfb (ZFN)	AGGTTGAGCAGGAAGCTCAG	AGGACTCGGACCCAGAGAAT
Cpt1	CTGAGACAGACTCACACCGCTT	GTTTTCCTTCCGTGTGGCTCAG
Fasn	TTGTGGACGGAGGTATCAACCC	CCATGCTGTAGCCCAGAAGAGT
Hprt1	TCAGTCCCAGCGTCGTGATTAG	TCGAGCAAGTCTTTCAGTCCTGT
Lep	CAGCAGCTGCAAGGTCCAAGA	TAGGACCAAAGCCACAGGAACC
Myh6	ACACCAACCTGTCCAAGTTCC	ATCGTGCATTTTCTGCTTGGCG
Myh7	CAACCTGTCCAAGTTCCGCAAG	ACTCTTCATTCAGGCCCTTGGC
Nppa	ATTTCAAGAACCTGCTAGACCACC	GCACCTCAGAGAGGGAGCTAAG
Nppb	ACAATCCACGATGCAGAAGCTG	GAAGGCGCTGTCTTGAGACCTA
Pgc1a	TTGACTGGCGTCATTCAGGAGC	CCAGGGCAGCACACTCTATGT
Renin	GATCACCATGAAGGGGGTCTCT	GATCAACTGCAGGGAGCTGGTA
Slc2a4	TTTGCACACCACTTCCGAAGGC	GGTTCCCCATCTTCAGAGCCGAT
Ucp1	ACATACTGGCAGATGACGTCCC	GCTGGGTACACTTGGGTACTGT

Table S3. Trait terms from the NHGRI-EBI GWAS catalog that were used to identify SNPs associated with cardiometabolic traits in the *CFB* locus

**NHGRI-EBI genome-wide association cardio-metabolic trait**
Basal_metabolic_rate
Blood_pressure
Blood_pressure_(age_interaction)
Blood_pressure_(anthropometric_measures_interaction)
Blood_pressure_(smoking_interaction)
Cardiac_hypertrophy
Cardiovascular_disease_in_hypertension_(ACE_inhibitor_interaction)
Cardiovascular_disease_in_hypertension_(calcium_channel_blocker_interaction)
Cardiovascular_disease_risk_factors
Cardiovascular_heart_disease_in_diabetics
Cholesterol
Cholesterol_and_Triglycerides
Cholesterol,_total
Coronary_heart_disease
Coronary_heart_disease_event_reduction_in_response_to_statin_therapy_(interaction)
Diabetes_related_insulin_traits
Diastolic_blood_pressure
Diastolic_blood_pressure_(alcohol_consumption_interaction)
Fasting_glucose-related_traits
Fasting_glucose-related_traits_(interaction_with_BMI)
Fasting_insulin_(interaction)
Fasting_insulin-related_traits
Fasting_insulin-related_traits_(interaction_with_BMI)
Fasting_plasma_glucose
Fasting_plasma_glucose_(childhood)
Glucose_homeostasis_traits
Glycemic_traits
HDL_cholesterol
HDL_Cholesterol_-_Triglycerides_(HDLC-TG)
Hypertension
Insulin_resistance/response
LDL_cholesterol
Lipoprotein_(a)_-_cholesterol_levels
Lipoprotein_(a)_levels
Metabolic_syndrome
Metabolic_traits
Systolic_blood_pressure
Systolic_blood_pressure_(alcohol_consumption_interaction)
Systolic_blood_pressure_in_sickle_cell_anemia
Triglycerides

Triglycerides-Blood_Pressure_(TG-BP)
Two-hour_glucose_challenge
Type_2_diabetes
Type_2_diabetes_(dietary_heme_iron_intake_interaction)
Type_2_diabetes_(young_onset)_and_obesity
Type_2_diabetes_and_gout
Type_2_diabetes_and_other_traits
Type_2_diabetes_nephropathy
Visceral_adipose_tissue
Visceral_adipose_tissue_adjusted_for_BMI
Visceral_adipose_tissue/subcutaneous_adipose_tissue_ratio
Visceral_fat

Table S4. Serum analytes

**Analyte**	**SHR**	***Cfb*** $^{-/-}$
Cholesterol (mM)	1.62 ± 0.05	1.26 ± 0.06***
Triglyceride (mM)	0.28 ± 0.01	0.24 ± 0.02**
Adiponectin (total) (ng/mL)	38.3 ± 2.8	43.4 ± 2.6
Adiponectin (HMW*) (ng/mL)	3.81 ± 0.14	2.36 ± 0.05***
Leptin (ng/mL)	0.95 ± 0.08	0.95 ± 0.05
Aldosterone (ng/mL)	272 ± 14	150 ± 6***
IL-2 (pg/mL)	undetected	undetected
IL-6 (pg/mL)	108.7 ± 6.4	undetected
IL-10 (pg/mL)	182.2 ± 24.6	45.9 ± 17.9*
GM-CSF† (pg/mL)	19.25 ± 4.9	10.5 ± 2.2
IFN-γ (pg/mL)	18.2 ± 1.1	7.12 ± 0.3***
TNFα (pg/mL)	8.05 ± 1.98	undetected

Results are mean ± SEM; **P* < 0.05, ***P* < 0.005, ****P* < 0.0001.
*HMW, high molecular weight.
†GM-CSF, granulocyte macrophage colony-stimulating factor.

Table S5. Left ventricle echocardiographic measurements at 7 weeks of age

**Parameter**	**SHR**	***Cfb*** $^{-/-}$
LV* Mass; d (mg)	646 ± 29	542 ± 43
LV (mg/kg)	4500 ± 154	3649 ± 268*
Endocardial Volume; d† (µL)	251 ± 14	248 ± 10
Endocardial Volume; s‡ (µL)	85 ± 9	64 ± 4
Endocardial Area Change (mm^2)	29.1 ± 1.5	33.6 ± 1.8
LV wall thickness; d (mm)	1.24 ± 0.05	1.08 ± 0.06
Heart Rate (beats/min)	324 ± 6	315 ± 7
Endocardial Stroke Volume (µL)	165 ± 10	183 ± 9
Ejection fraction (%)	66.2 ± 2.3	73.9 ± 1.7*
Fractional area change (%)	47.0 ± 1.8	54.8 ± 1.9**
Fractional shortening (%)	36.1 ± 0.6	43.4 ± 1.0**
Cardiac output (mL/min)	53.9 ± 3.5	57.6 ± 2.5

Results are mean ± SEM; *$P < 0.05$, **$P < 0.005$, ***$P < 0.0001$.
*left ventricle.
†d, diastole.
‡s, systole.

Table S6. NHGRI-EBI cardio-metabolic GWAS hits located at the *CFB* locus

**Disease/trait**	**Strongest SNP/ risk allele**	**Chromosome position**	**Distance from *Cfb* (Mb)**
Type 2 diabetes	rs3132524-G	31168937	0.775
Coronary heart disease	rs3869109-G	31216419	0.728
LDL cholesterol, total cholesterol	rs9357121	31272702	0.671
Triglycerides	rs2247056-T	31297713	0.646
SBP, DBP	rs9266359-C	31364962	0.579
Type 2 diabetes	rs2244020-G	31379674	0.564
Visceral fat adjusted for BMI	rs12175489-A	31409810	0.534
Metabolic syndrome	rs3099844-A	31481199	0.463
SBP, DBP, Hypertension	rs805303-G	31648589	0.296
SBP, DBP, Hypertension	rs2021783-C	32077074	0.126
Triglycerides	rs419132-G	32243022	0.292
Visceral fat	rs13196329-C	32357594	0.407
Coronary heart disease	rs9268402-G	32373576	0.423
Cholesterol, total	rs3177928-A	32444658	0.494
Cholesterol, total	rs114067101-G	32490183	0.539
HDL cholesterol	rs116569761	32680379	0.729
Coronary heart disease	rs11752643-T	32701596	0.751
Type 2 diabetes	rs3916765-A	32717773	0.767

Table S7. GTex *cis*-eQTLs associated with *CFB* expression

SNP Id	P-value	Effect size	Tissue	Chromosome position (Hg38)	Distance from TSS*
rs115056371	0.000084	0.17	Adipose_Subcutaneous	31238942	-706731
chr6_32630981_D	0.000051	0.18	Adipose_Subcutaneous	32663204	717531
rs9274179	0.000054	0.18	Adipose_Subcutaneous	32662687	717014
rs28746813	0.000065	0.18	Adipose_Subcutaneous	32665453	719780
chr6_32656068_I	0.000072	0.18	Adipose_Subcutaneous	32688291	742618
rs28746811	0.000076	0.18	Adipose_Subcutaneous	32665420	719747
rs28746814	0.000085	0.18	Adipose_Subcutaneous	32665470	719797
rs116066079	0.0001	0.18	Adipose_Subcutaneous	32712646	766973
rs114682366	0.0001	0.18	Adipose_Subcutaneous	32712664	766991
rs28724263	0.000023	0.19	Adipose_Subcutaneous	32664152	718479
rs114830099	0.000028	0.19	Adipose_Subcutaneous	32742444	796771
rs114515571	0.000041	0.19	Adipose_Subcutaneous	32713384	767711
rs114227315	0.000041	0.19	Adipose_Subcutaneous	32712602	766929
rs9274657	0.0000045	0.2	Adipose_Subcutaneous	32668587	722914
rs9274659	0.0000045	0.2	Adipose_Subcutaneous	32668608	722935
chr6_32656067_I	0.000021	0.2	Adipose_Subcutaneous	32688290	742617
rs9274209	0.000038	0.2	Adipose_Subcutaneous	32663043	717370
rs28746806	0.000043	0.2	Adipose_Subcutaneous	32665288	719615
rs28746832	0.000005	0.21	Adipose_Subcutaneous	32666039	720366
chr6_32632717	0.000049	0.22	Adipose_Subcutaneous	32664940	719267
rs9274227	0.000059	0.22	Adipose_Subcutaneous	32663365	717692
rs191863247	0.0000039	0.27	Adipose_Subcutaneous	32487582	541909

chr6_32632878_I	0.000042	0.28	Adipose_Subcutaneous	32665101	719428
chr6_32627913_D	0.000056	0.39	Adipose_Subcutaneous	32660136	714463
rs60302302	0.0000064	0.41	Adipose_Subcutaneous	32515926	570253
rs181165562	0.000075	0.41	Adipose_Subcutaneous	32386129	440456
rs76846904	0.000015	0.78	Adipose_Subcutaneous	32532140	586467
rs76415507	0.000009	-0.4	Artery_Aorta	32524812	579139
rs143726520	0.0000044	-0.36	Artery_Aorta	32520080	574407
rs114624824	0.000013	-0.34	Artery_Aorta	32524743	579070
rs74655967	0.000013	-0.34	Artery_Aorta	32524691	579018
rs115623335	0.0000036	-0.33	Artery_Aorta	32564801	619128
rs76851429	0.000041	-0.33	Artery_Aorta	32524591	578918
rs116640755	0.00002	-0.32	Artery_Aorta	32564779	619106
rs80237386	0.000027	-0.32	Artery_Aorta	32524716	579043
rs75906455	0.00003	-0.32	Artery_Aorta	32524742	579069
rs77159841	0.000038	-0.32	Artery_Aorta	32524733	579060
rs72492345	0.000049	-0.32	Artery_Aorta	32564838	619165
rs146763062	0.000027	-0.31	Artery_Aorta	32523894	578221
rs115814063	0.000039	-0.31	Artery_Aorta	32524316	578643
chr6_32551762_D	0.000062	-0.31	Artery_Aorta	32583985	638312
chr6_32490131_D	0.000065	-0.31	Artery_Aorta	32522354	576681
rs114553448	0.000083	-0.31	Artery_Aorta	32569362	623689
rs115918114	0.00005	-0.3	Artery_Aorta	32524524	578851
rs79949014	0.000086	-0.3	Artery_Aorta	32524609	578936
rs142399500	0.000059	-0.29	Artery_Aorta	32521691	576018
rs141142229	0.000082	-0.29	Artery_Aorta	32524028	578355

rs114980010	0.0000041	-0.33	Artery_Tibial	31604704	-340969
rs1048709	0.000049	-0.25	Artery_Tibial	31947158	1485
rs115804811	0.0000022	-0.81	Skin_Sun_Exposed_Lower_leg	32570025	624352
rs74216018	0.0000089	-0.47	Skin_Sun_Exposed_Lower_leg	32524667	578994
rs34382076	0.00001	-0.44	Skin_Sun_Exposed_Lower_leg	32581548	635875
rs79606458	0.000045	-0.28	Skin_Sun_Exposed_Lower_leg	32522036	576363

*TSS, transcription start site

Table S8. Genes residing in the 1 MB region upstream/downstream the *CFB* transcription start site

Gene stable ID	Gene Start (bp)	Gene End (bp)	Gene name
ENSG00000233529	30945979	30954862	HCG21
ENSG00000275906	30961403	30962396	XXbac-BPG118E17.10
ENSG00000204544	30983718	30989903	MUC21
ENSG00000261272	31010474	31035402	MUC22
ENSG00000228789	31053450	31059890	HCG22
ENSG00000222895	31083010	31083109	RNU6-1133P
ENSG00000204542	31111223	31112559	C6orf15
ENSG00000204540	31114750	31140092	PSORS1C1
ENSG00000204539	31115090	31120446	CDSN
ENSG00000204538	31137536	31139350	PSORS1C2
ENSG00000238211	31140727	31140913	POLR2LP1
ENSG00000204536	31142439	31158238	CCHCR1
ENSG00000137310	31158542	31167159	TCF19
ENSG00000204531	31164337	31180731	POU5F1
ENSG00000204528	31173735	31177899	PSORS1C3
ENSG00000272501	31195200	31198037	XXbac-BPG299F13.17
ENSG00000206344	31197760	31203968	HCG27
ENSG00000271821	31200165	31201918	XXbac-BPG299F13.14
ENSG00000255726	31222913	31223093	XXbac-BPG299F13.15
ENSG00000255899	31224342	31225058	XXbac-BPG299F13.16
ENSG00000204525	31268749	31272130	HLA-C
ENSG00000234745	31269491	31357188	HLA-B
ENSG00000214892	31275572	31278754	USP8P1
ENSG00000227939	31280317	31281519	RPL3P2
ENSG00000231402	31287510	31288964	WASF5P
ENSG00000256166	31293908	31301642	XXbac-BPG248L24.13
ENSG00000229836	31307815	31308549	XXbac-BPG248L24.10
ENSG00000277402	31355224	31355316	MIR6891
ENSG00000271581	31356647	31357637	XXbac-BPG248L24.12

ENSG00000228432	31366352	31366898	DHFRP2
ENSG00000201658	31370134	31370240	RNU6-283P
ENSG00000230994	31377419	31378019	FGFR3P1
ENSG00000223702	31380411	31380839	ZDHHC20P2
ENSG00000225851	31382074	31382288	HLA-S
ENSG00000272221	31394289	31395495	XXbac-BPG181B23.7
ENSG00000204520	31399784	31415315	MICA
ENSG00000206337	31400702	31477506	HCP5
ENSG00000199332	31402152	31402250	Y_RNA
ENSG00000230174	31441667	31446973	LINC01149
ENSG00000233902	31462728	31463336	XXbac-BPG181B23.6
ENSG00000204516	31494881	31511124	MICB
ENSG00000201680	31496689	31496790	Y_RNA
ENSG00000256851	31515979	31516211	XXbac-BPG16N22.5
ENSG00000219797	31519480	31520291	PPIAP9
ENSG00000225499	31528114	31528693	RPL15P4
ENSG00000204511	31528717	31530232	MCCD1
ENSG00000198563	31530219	31542448	DDX39B
ENSG00000254870	31530219	31546608	ATP6V1G2-DDX39B
ENSG00000201785	31536374	31536449	SNORD117
ENSG00000265236	31541101	31541178	SNORD84
ENSG00000234006	31542304	31543138	DDX39B-AS1
ENSG00000213760	31544462	31548427	ATP6V1G2
ENSG00000204498	31546870	31558829	NFKBIL1
ENSG00000226979	31572054	31574324	LTA
ENSG00000232810	31575567	31578336	TNF
ENSG00000227507	31580525	31582522	LTB
ENSG00000204482	31586124	31588909	LST1
ENSG00000204475	31588895	31592985	NCR3
ENSG00000230622	31611083	31611356	UQCRHP1
ENSG00000204472	31615184	31617021	AIF1

ENSG00000204469	31620720	31637771	PRRC2A
ENSG00000200816	31623079	31623210	SNORA38
ENSG00000274494	31633787	31633858	MIR6832
ENSG00000204463	31639028	31652705	BAG6
ENSG00000204444	31652416	31658210	APOM
ENSG00000204439	31658298	31660772	C6orf47
ENSG00000227198	31658329	31660721	C6orf47-AS1
ENSG00000204438	31661229	31666283	GPANK1
ENSG00000201207	31663288	31663401	Y_RNA
ENSG00000204435	31665236	31670343	CSNK2B
ENSG00000263020	31666102	31673546	XXbac-BPG32J3.22
ENSG00000240053	31670167	31673776	LY6G5B
ENSG00000204428	31676684	31684040	LY6G5C
ENSG00000204427	31686949	31703444	ABHD16A
ENSG00000204422	31686962	31714072	XXbac-BPG32J3.20
ENSG00000266776	31701029	31701091	MIR4646
ENSG00000204424	31706885	31710595	LY6G6F
ENSG00000250641	31706904	31717918	XXbac-BPG32J3.19
ENSG00000255552	31711771	31714065	LY6G6E
ENSG00000244355	31715356	31717804	LY6G6D
ENSG00000204420	31718594	31726714	MPIG6B
ENSG00000204421	31718648	31721845	LY6G6C
ENSG00000213722	31727038	31730617	DDAH2
ENSG00000213719	31730581	31739763	CLIC1
ENSG00000204410	31739948	31762834	MSH5
ENSG00000255152	31740020	31764851	MSH5-SAPCD1
ENSG00000252743	31756951	31757053	RNU6-850P
ENSG00000228727	31762799	31764851	SAPCD1
ENSG00000235663	31764310	31765588	SAPCD1-AS1
ENSG00000204396	31765590	31777294	VWA7
ENSG00000204394	31777518	31795953	VARS

ENSG00000201555	31778817	31778905	Y_RNA
ENSG00000204392	31797396	31806984	LSM2
ENSG00000204390	31809619	31815065	HSPA1L
ENSG00000204389	31815464	31817946	HSPA1A
ENSG00000204388	31827735	31830255	HSPA1B
ENSG00000204387	31834608	31839766	C6orf48
ENSG00000201823	31835263	31835326	SNORD48
ENSG00000201754	31837076	31837142	SNORD52
ENSG00000204386	31857659	31862906	NEU1
ENSG00000204385	31863192	31879046	SLC44A4
ENSG00000204371	31879759	31897687	EHMT2
ENSG00000237080	31883761	31884204	EHMT2-AS1
ENSG00000166278	31897785	31945672	C2
ENSG00000204366	31899607	31901992	ZBTB12
ENSG00000244255	31927698	31952048	XXbac-BPG116M5.17
ENSG00000281756	31934474	31941724	C2-AS1
ENSG00000243649	31945650	31952084	CFB
ENSG00000204356	31952087	31959110	NELFE
ENSG00000284446	31956839	31956940	MIR1236
ENSG00000204351	31959080	31969755	SKIV2L
ENSG00000204348	31969810	31972292	DXO
ENSG00000204344	31971091	31982821	STK19
ENSG00000244731	31982024	32002681	C4A
ENSG00000233627	31999976	32003521	C4A-AS1
ENSG00000204338	32005636	32008451	CYP21A1P
ENSG00000248290	32008614	32012472	TNXA
ENSG00000250535	32013270	32013787	STK19B
ENSG00000224389	32014762	32035418	C4B
ENSG00000229776	32032713	32036258	C4B-AS1
ENSG00000231852	32038265	32041670	CYP21A2
ENSG00000168477	32041154	32115334	TNXB

ENSG00000252512	32078508	32078628	RNA5SP206
ENSG00000213676	32098176	32128253	ATF6B
ENSG00000204315	32128707	32130291	FKBPL
ENSG00000204314	32148359	32154373	PRRT1
ENSG00000221988	32153441	32163680	PPT2
ENSG00000258388	32153845	32171978	PPT2-EGFL8
ENSG00000241404	32164583	32168281	EGFL8
ENSG00000204310	32168212	32178096	AGPAT1
ENSG00000284469	32170030	32170116	MIR6721
ENSG00000204308	32178354	32180793	RNF5
ENSG00000277264	32179816	32179876	MIR6833
ENSG00000204305	32180968	32184324	AGER
ENSG00000273333	32184733	32185882	XXbac-BPG300A18.13
ENSG00000204304	32184741	32190186	PBX2
ENSG00000213654	32190766	32195523	GPSM3
ENSG00000204301	32194843	32224067	NOTCH4
ENSG00000277427	32255284	32350039	XXbac-BPG154L12.5
ENSG00000225914	32255711	32265838	XXbac-BPG154L12.4
ENSG00000204296	32288526	32371912	C6orf10
ENSG00000237285	32325219	32326178	HNRNPA1P2
ENSG00000223335	32352877	32352983	RNU6-603P
ENSG00000228962	32390510	32393686	HCG23
ENSG00000204290	32393963	32407128	BTNL2
ENSG00000204287	32439842	32445046	HLA-DRA
ENSG00000196301	32459821	32473500	HLA-DRB9
ENSG00000198502	32517343	32530287	HLA-DRB5
ENSG00000251916	32549940	32550090	RNU1-61P
ENSG00000229391	32552713	32560022	HLA-DRB6
ENSG00000196126	32578769	32589848	HLA-DRB1
ENSG00000196735	32628179	32647062	HLA-DQA1
ENSG00000179344	32659467	32668383	HLA-DQB1

ENSG00000223534	32659880	32660729	HLA-DQB1-AS1
ENSG00000235040	32706124	32706955	MTCO3P1
ENSG00000232080	32718005	32719170	XXbac-BPG254F23.7
ENSG00000226030	32730758	32731695	HLA-DQB3
ENSG00000237541	32741342	32747215	HLA-DQA2
ENSG00000263649	32749912	32749979	MIR3135B
ENSG00000232629	32756098	32763534	HLA-DQB2
ENSG00000241106	32812763	32817048	HLA-DOB
ENSG00000250264	32813767	32838822	XXbac-BPG246D15.9
ENSG00000204267	32821833	32838780	TAP2
ENSG00000204264	32840717	32844703	PSMB8
ENSG00000204261	32844086	32846495	PSMB8-AS1
ENSG00000240065	32844136	32859585	PSMB9
ENSG00000168394	32845209	32853978	TAP1
ENSG00000234515	32879171	32879848	PPP1R2P1
ENSG00000235301	32896416	32896490	HLA-Z
ENSG00000242574	32934629	32941070	HLA-DMB

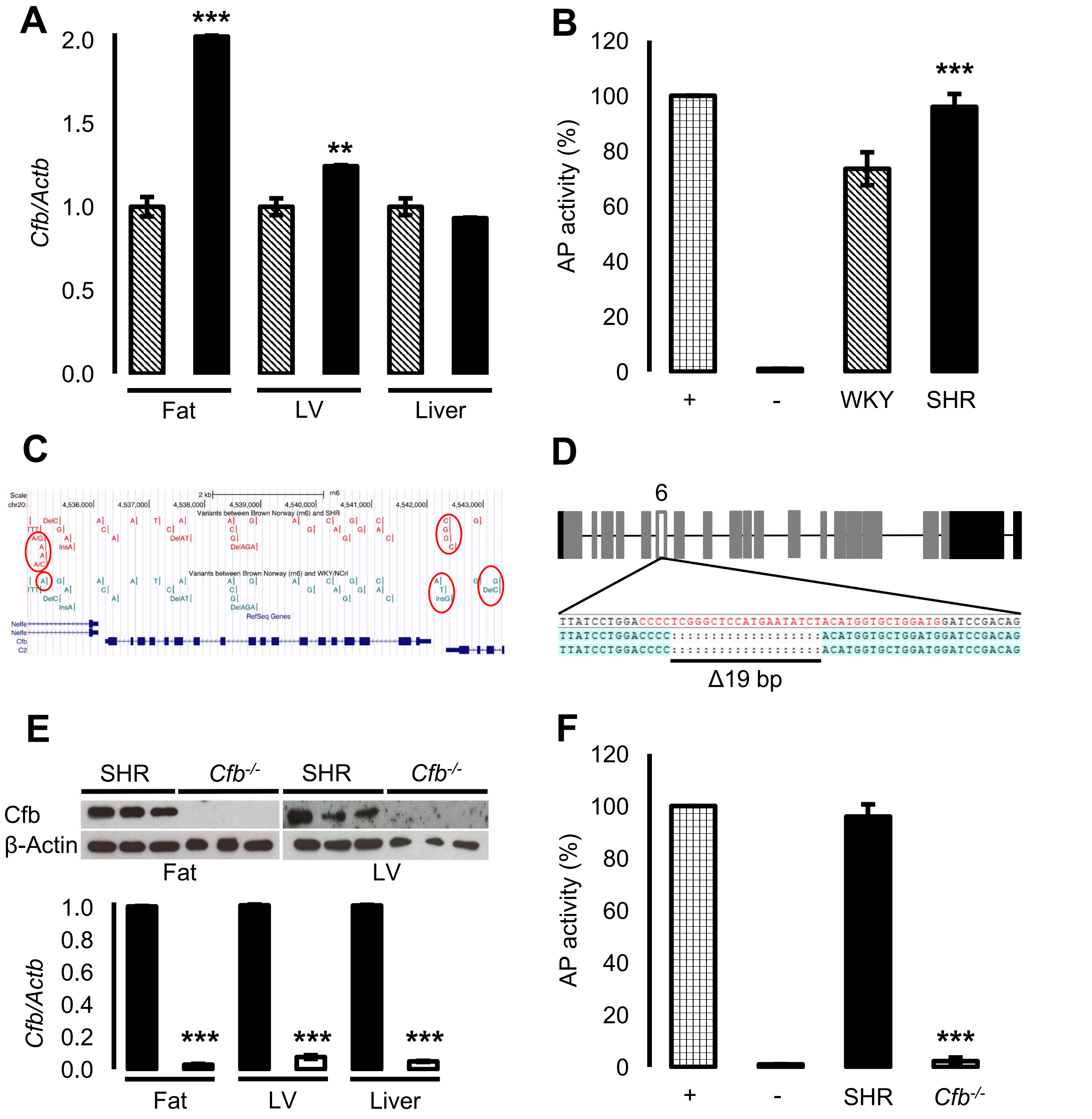

Figure S1. Generation of a complement factor b knockout rat on an SHR background. (A) qPCR analysis of *Cfb* expression epididymal (Fat), left ventricle (LV) and liver from SHR (filled bars) and WKY (striped bars). (B) Serum alternative complement (AP) activity in SHR compared to WKY (+, positive control, -, negative control). (C) Graphical representation of *Cfb* detailing unique variants in SHR (red-circled) compared to BN and WKY. (D) Diagram of the exon-intron structure of the rat *Cfb* gene indicating the 19 bp deletion generated by zinc-finger nucleases in exon 6. (E) qPCR analysis of *Cfb* and immunoblot of Cfb protein expression in epididymal adipose tissue (Fat), left ventricle (LV) and liver, showing protein and transcript ablation in *Cfb*$^{-/-}$ tissues, SHR (black-filled bars) and *Cfb*$^{-/-}$ (white-filled bars). (F) Serum AP complement activity in *Cfb*$^{-/-}$ (open bar) compared to SHR (filled bar) (+, positive control, -, negative control). (n = 5-6 per group). *P <0.05, **P <0.01, ***P <0.001.

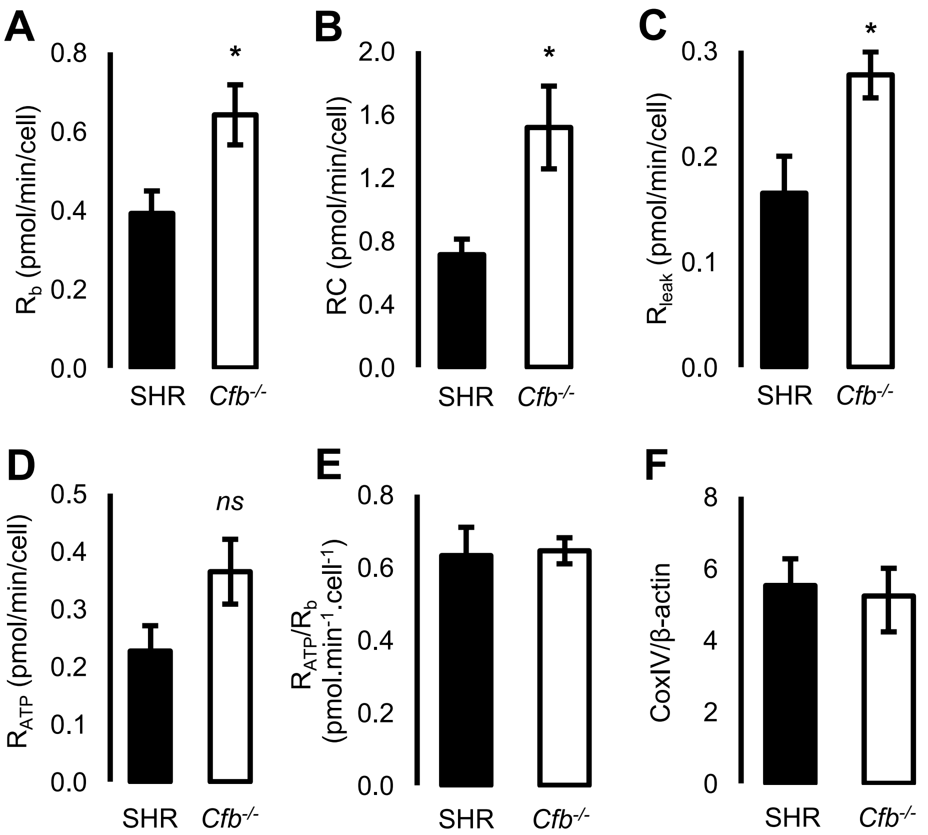

Figure S2. Oxygen consumption rate (OCR) and CoxIV abundance in isolated adipocytes from SHR and *Cfb*$^{-/-}$ rats. (A) basal respiratory rate, (B) reserve capacity (RC), (C) leak respiration, (D) ATP-linked respiration, and (E) ATP efficiency non-respiratory oxygen consumption rate in isolated epididymal adipocytes. (F) expression of CoxIV protein abundance in epididymal fat (n = 6 per group). *P <0.05.

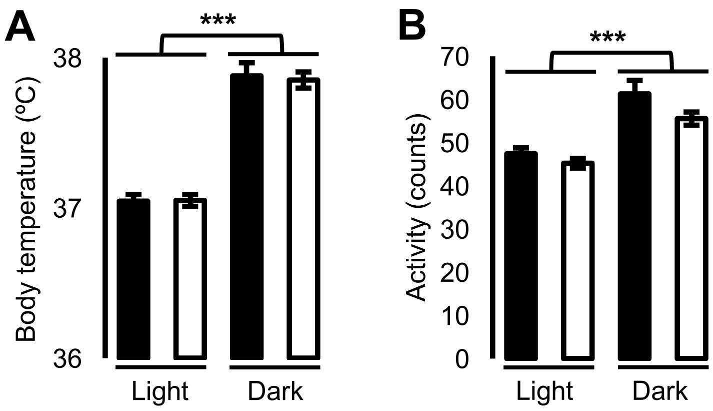

Figure S3. Telemetric measurements of (A) mean core body temperature and (B) activity (n = 8-9 per group). *Cfb*$^{-/-}$ (open bars) compared to SHR (filled bars). Significant differences between light and dark periods ***P <0.001.

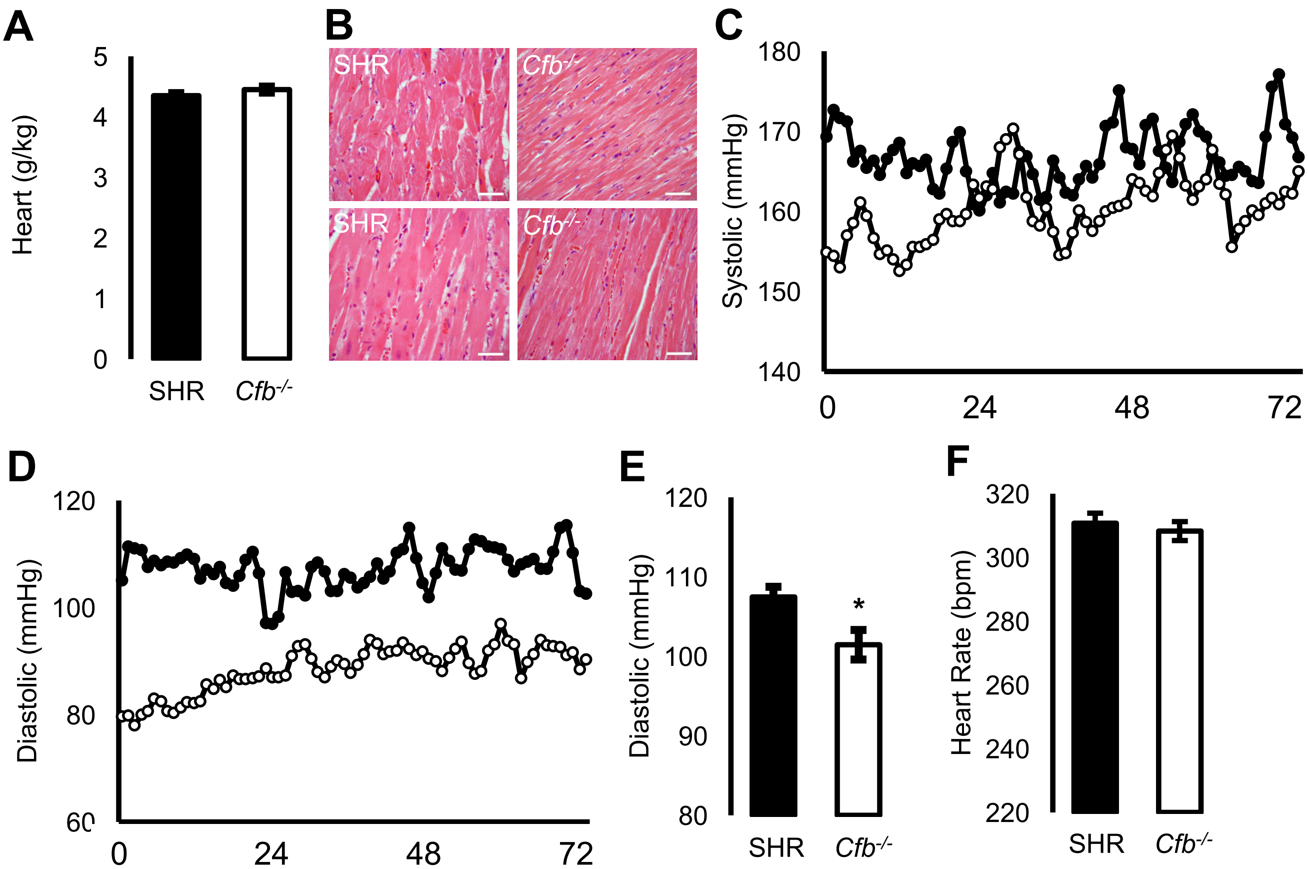

Figure S4. Baseline cardiovascular measurements. (A) Relative heart wet mass (n = 15 per group). (B) Light micrographs of representative H&E stained left ventricle sections (scale bar 10 µm). (C) Systolic and (D) diastolic blood pressure hourly plots during 72 h (n = 8-9 per group). (E) Mean diastolic blood pressure and (G) Heart rate. $^*P<0.05$.

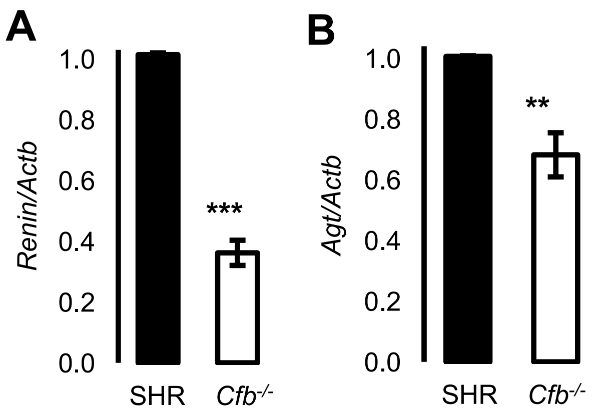

Figure S5. Gene expression of (H) renal renin and (I) hepatic angiotensinogen (n = 6 per group). ***P* <0.01, ****P* <0.001

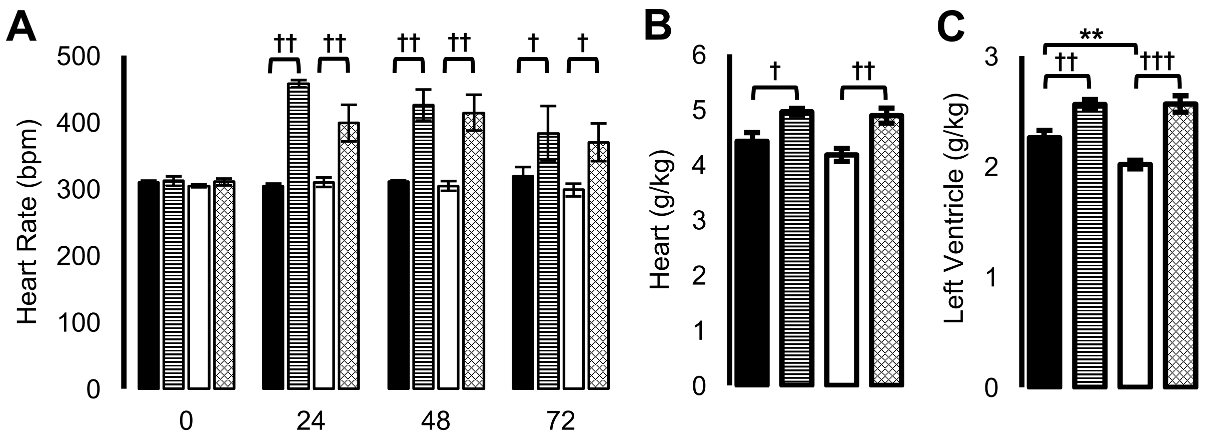

Figure. S6. Wet cardiac masses taken from rats treated with isoproterenol and saline for 72 h. (A) Heart rate, (B) relative heart and (C) left ventricle wet masses (n = 4-5 per group). Black-filled bars, SHR, saline-treated; Stripe-filled bars, SHR, isoproterenol-treated; White-filled bars, *Cfb*$^{-/-}$, saline-treated; Hatch-filled bars, *Cfb*$^{-/-}$, isoproterenol-treated. Differences in genotype ***P* <0.01 or treatment $^{\dagger}P$ <0.05, $^{\dagger\dagger}P$ <0.01, $^{\dagger\dagger\dagger}P$ <0.001 .

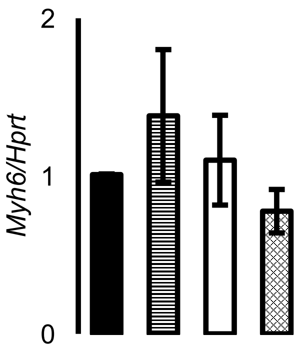

Figure S7. *Myh6* expression levels in left ventricles following 72h isoproterenol or saline treatment. Black-filled bars, SHR, saline-treated; Stripe-filled bars, SHR, isoproterenol-treated; White-filled bars, *Cfb*$^{-/-}$, saline-treated; Hatch-filled bars, *Cfb*$^{-/-}$, isoproterenol-treated.
